# Anti-S-layer monoclonal antibodies impact *Clostridioides difficile* physiology

**DOI:** 10.1080/19490976.2023.2301147

**Published:** 2024-01-30

**Authors:** Lise Hunault, Emile Auria, Patrick England, Julien Deschamps, Romain Briandet, Vanessa Kremer, Bruno Iannascoli, Léo Vidal-Maison, Chunguang Guo, Lynn Macdonald, Séverine Péchiné, Cécile Denève-Larrazet, Bruno Dupuy, Guy Gorochov, Pierre Bruhns, Delphine Sterlin

**Affiliations:** a Centre d’Immunologie et des Maladies Infectieuses (CIMI-Paris), Sorbonne Université, Inserm, CNRS, Paris, France; b Antibodies in Therapy and Pathology, Institut Pasteur, Université Paris-Cité, Inserm UMR1222, Paris, France; c Collège doctoral, Sorbonne Université, Paris, France; d Laboratoire Pathogenèse des Bactéries Anaérobies, Institut Pasteur, Université Paris-Cité, UMR-CNRS 6047, Paris, France; e Department of Structural Biology and Chemistry, Institut Pasteur, Université Paris Cité, CNRS UMR3528, Plateforme de Biophysique Moléculaire, Paris, France; f Institut Micalis, Université Paris-Saclay, INRAE, AgroParisTech, Jouy-en-Josas, France; g Inflammation, Microbiome and Immunosurveillance, Université Paris-Saclay, Inserm, Châtenay-Malabry, France; h Regeneron Pharmaceuticals, Tarrytown, NY, USA; i Equipe Bactéries Pathogènes et Santé, Faculté de Pharmacie, Institut MICALIS (UMR 1319 Université Paris-Saclay, INRAE, AgroParisTech), Orsay, France

**Keywords:** *Clostridioides difficile*, monoclonal antibodies, S-layer, growth, biofilms, toxins, neutrophils

## Abstract

*Clostridioides difficile* (*C. difficile*), a gram-positive anaerobic and spore-forming bacterium, is the leading cause of nosocomial antibiotic-associated diarrhea in adults which is characterized by high levels of recurrence and mortality. Surface (S)-layer Protein A (SlpA), the most abundantly expressed protein on the bacterial surface, plays a crucial role in the early stages of infection although the nature of its involvement in *C. difficile* physiology is yet to be fully understood. Anti-S-layer antibodies have been identified in the sera of convalescent patients and have been correlated with improved outcomes of *C. difficile* infection (CDI). However, the precise mechanisms by which anti-S-layer antibodies confer protection to the host remain unknown. In this study, we report the first monoclonal antibodies (mAbs) targeting the S-layer of reference strain 630. Characterization of these mAbs unraveled important roles for the S-layer protein in growth, toxin secretion, and biofilm formation by *C. difficile*, with differential and even opposite effects of various anti-SlpA mAbs on these functions. Moreover, one anti-SlpA mAb impaired *C. difficile* growth and conferred sensitivity to lysozyme-induced lysis. The results of this study show that anti-S-layer antibody responses can be beneficial or harmful for the course of CDI and provide important insights for the development of adequate S-layer-targeting therapeutics.

## Introduction

*Clostridioides difficile* is an anaerobic, gram-positive, spore-forming bacterium that is the leading agent responsible for nosocomial antibiotic-associated diarrhea and colitis in adults.^1^ *C. difficile* infection (CDI) causes substantial morbidity and mortality with severe pseudomembranous colitis, which is characterized by extensive colonic damage and intestinal inflammation. While CDI symptoms have largely been attributed to bacterial toxins, there is growing attention on the possible involvement of *C. difficile* adhesins and surface proteins in gut colonization and evasion of immune system surveillance. The latter proteins play a major role in triggering bacterial pathogenesis by interacting with Toll-Like Receptor 4 (TLR4) and inducing an inflammatory response.^1,2^ Among these proteins, *C. difficile* Surface (S)-layer protein A (SlpA) has gained substantial interest.

The *C. difficile* S-layer is composed of two main proteins *i.e.*, the High-Molecular Weight (HMW) and the Low-Molecular Weight (LMW) Surface Layer Proteins (SLPs) that derive from the common SlpA precursor. SlpA is first secreted and then cleaved by the cell wall cysteine protease Cwp84, releasing the two mature subunits, HMW and LMW. These two subunits associate to form a stable heterodimeric complex, which is anchored to the cell wall by the HMW, with the LMW being the most external subunit. SlpA is secreted throughout the cytoplasmic membrane and constitutes an interwall reservoir that fills the gaps formed during growth or damage.^3^ With the assembly of the S-layer in the areas of newly synthesized peptidoglycan, *C. difficile* can maintain a stable S-layer that continuously protects the cell. One astonishing characteristic of the *C. difficile* S-layer is its compactness. With pores of only 10 Å in diameter, it is more compact than S-layers of other bacterial species, whose pores range from 30 Å to 100 Å. This renders *C. difficile* impermeable to large molecules such as lysozyme^4^ to which it is resistant.

The S-layer is crucial for bacterial integrity and *C. difficile* S-layer-null mutants display severely impaired physiological functions. They are highly sensitive to innate immune effector molecules, such as lysozyme, show sporulation defects, and produce fewer toxins *in vitro* than wild type strains.^5^ *C. difficile* persistence and recurrence have been linked to the presence of spores^6^ and might also be associated with its ability to form biofilms in the gut.^7^ Biofilm formation constitutes a process during which microorganisms adopt a multicellular behavior in a thick enclosed matrix composed of extracellular polymeric substances that facilitates and/or prolongs survival in diverse environmental niches.^8^ Cwp84 mutants with an altered S-layer display increased biofilm generation, suggesting that intact S-layers prevent aggregation, which is one of the first steps to generate biofilms.^9^ As the predominant surface protein, the *C. difficile* S-layer has also been implicated in the attachment to intestinal cells both *in vitro* and *ex vivo*.^10,11^

The S-layer is immunogenic, as anti-SLP antibodies have been detected in the sera of convalescent patients, and are associated with improved CDI outcomes.^12,13^ In animal models, passive immunization using anti-SlpA serum delayed *C. difficile* colonization in mice,^14^ whereas active immunization with recombinant SlpA slightly prolonged the survival of hamsters infected with *C. difficile*.^15^ In addition, anti-LMW nanobodies have been shown to decrease bacterial motility *in vitro*.^16^ However, the extent to which anti-S-layer humoral responses interfere with *C. difficile* fitness and CDI pathogenesis remains unclear. In this respect, no monoclonal antibodies (mAbs) targeting the S-layer that can be used to explore the role of SlpA *in vivo* have been reported to date.

Here, we generated and characterized a first series of anti-LMW mAbs to study S-layer interactions with the host immune response. We describe the differential effects of anti-LMW mAbs on *C. difficile* physiology in terms of growth, toxin secretion, and biofilm formation *in vitro*. Our work deciphers the interactions between antibodies and various epitopes of the S-layer with unexpectedly different outcomes and further describes the role of *C. difficile* S-layer in bacterial fitness.

## Results

### Generation and characterization of high-affinity LMW-specific mAbs

**Figure 1.**
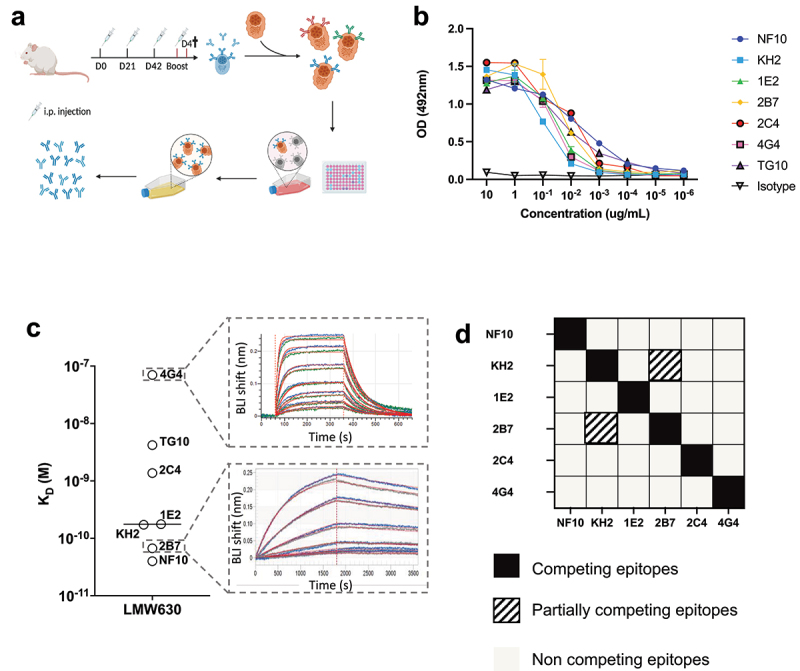
High-affinity anti-LMW mAbs bind distinct epitopes a. Schematic view of immunization, hybridoma generation and screening for obtention of anti-LMW mAbs. b. Mab binding to recombinant LMW measured by ELISA at indicated concentrations. Dark curve represents isotype control. c. Affinities toward LMW determined by Bio-Layer Interferometry. Representative sensorgrams of one low (4G4) and one high-affinity (2B7) mAb. Antibody concentration from 500 nM to 8 nM for 4G4 and from 2 nM to 0.02 nM for 2B7 were tested, as shown from top to bottom. Blue curves represent raw data while red curves represent fitting with a 1:1 antibody:antigen model. d. Summary table representing the results of BLI-based competitive of anti-LMW mAbs toward LMW.

To investigate the role of the S layer in *C. difficile* biology, we generated a collection of mAbs targeting the SlpA LMW, the most external subunit of the S-layer, of the reference strain *C. difficile* 630Δerm (CD630Δ*erm*), a spontaneous erythromycin-sensitive derivative of reference strain 630. Because humanized anti-LMW antibodies may be of therapeutic interest for the treatment of CDIs, we used knock-in mice in which the endogenous genes encoding the heavy chain variable (VH) and light chain variable (VL) domains were replaced by their human counterparts (Velocimmune mice, Supplemental Figure 1a).^17,18^ Even though in this study we focused on mAbs produced with mouse heavy and light chain constant domain, it is of interest to note that cloning of these VH and VL into vectors containing human heavy and light chain constant domains allowed for the direct generation - *in fine* - of fully human anti-LMW mAbs. Velocimmune and BALB/c mice were immunized with recombinant LMW (Supplemental Figure 1b) on D0, D21, D42 and four days before spleen collection, according to the schedule shown in Figure 1a. Anti-LMW hybridomas were generated from the splenocytes of one Velocimmune and one BALB/c mouse using ELISA as a screening method (Figure 1a). Seven anti-LMW mAbs (all mouse IgG1, Supplemental Fig. s1c) demonstrated binding to the LMW at concentrations as low as 10^−2^ µg/mL in an anti-LMW ELISA. The mouse VH-VL sequence-containing mAbs NF10 and KH2, and the human VH-VL-sequence-containing mAbs 1E2, 2B7, 2C4 and 4G4 were generated from the BALB/C and Velocimmune mice, respectively.

Bio-layer interferometry (BLI) experiments revealed a very large range of equilibrium dissociation constants (K_D_), ranging from 32 pM to 70 nM, corresponding to low to very high affinity antibodies (Figure 1c). The mAb with the lowest affinity displayed a fast on/off profile with a high dissociation rate (k_off_) of ~ 0.01 s^−1^, whereas the two mAbs with the highest affinities displayed a very low k_off_ of ~ 0.00003 s^−1^ (Table S1). To examine whether anti-LMW mAbs recognize overlapping or distinct epitopes on LMW, we designed a competitive BLI assay, using a pre-bound anti-LMW Ab as a competitor. Only two mAbs, KH2 and 2B7, partially competed for binding to LMW (Figure 1d).

Taken together, using the hybridoma technique, we generated a series of mostly high-affinity anti-LMW mAbs that targeted five different and non-overlapping epitopes on *C. difficile* SlpA LMW-630.

### *Binding to* C. difficile *630 vegetative cells*

**Figure 2.**
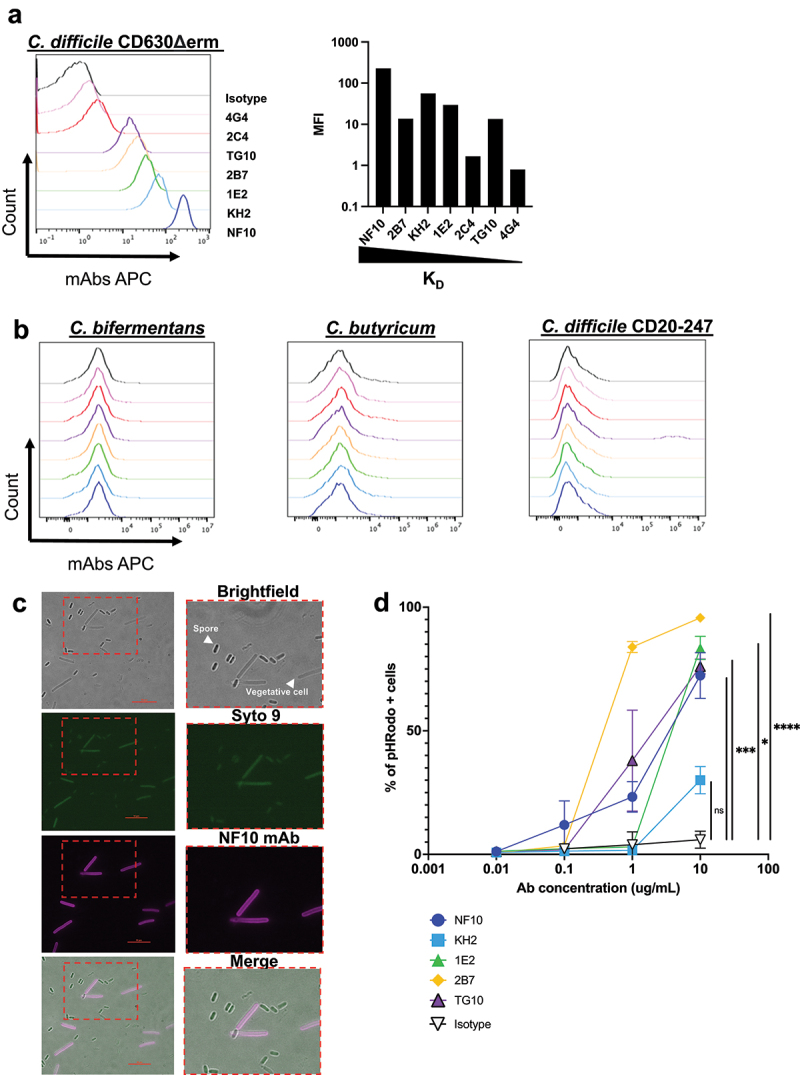
Anti-LMW mAbs bind vegetative *C. difficile* cells and enhance phagocytosis. a-b. Flow cytometry analysis of mAbs binding to indicated *C. difficile* reference strain 630 (a)and other *Clostridium* species or *C. difficile* strain (CD20–247 R012) (b). Black curve corresponds to isotype control. c. Representative view of mAb binding to *C. difficile* vegetative cells but not to spores. DNA from vegetative cells and spores was labeled with SYTO9 while mAb-coated bacteria were stained with AF647-conjugated anti-mouse IgG antibody. Merged staining was presented on the right panel. Analysis was performed by confocal microscopy. d. Percentage of neutrophils that have phagocytosed *C. difficile-*opsonized by the indicated mAb after 60 min and assessed by flow cytometry. Data represent mean + SEM of *n* = 3 technical replicates. Experiment was performed with at least two biological replicates. Asterisks indicate statistical significance with a two-way ANOVA test (ns: not significant; *p <.05, **p <.01, *** *p* <.001, and ****p <.0001).

Since LMW is the most exposed S-layer protein of *C. difficile*, we next assessed mAb binding to *C. difficile* whole bacteria. For this purpose, we used a previously reported bacterial flow cytometric assay.^17^ Five out of the seven anti-LMW mAbs readily bound CD630Δ*erm* (median fluorescence intensity (MFI) 100- to 1,000-fold higher than that of the isotype control). Binding of mAbs 2C4 and 4G4 to *C. difficile* strain 630 was much reduced (MFI 5 and 2.5-fold higher, respectively, than the isotype control, Figure 2a). These results are in agreement with the affinities of these mAbs for LMW, with mAb 4G4 possessing the worst affinity (70 nM). MAb 2C4, however, is expected to bind *C. difficile* under these conditions (K_D_ = 1.37 nM, Figure 2a), but its epitope may be partially inaccessible. In addition, the high affinity mAb 2B7, (K_D_ = 67pM), displayed only moderate binding, 10 times lower than that of NF10, which displayed a similar affinity for LMW (K_D_ = 43pM). None of these seven mAbs cross-reacted with commensal bacteria of the same genus, *i.e. Clostridium bifermentans* and *Clostridium butyricum*, confirming their *C. difficile* specificity (Figure 2b, left and middle panel). In addition, there was no cross-reactivity with a different ribotype (012) of the *C. difficile* strain CD20–247, which is consistent with the low inter-strain homology of the LMWs (Figure 2b, right panel).

In this part, we show that most of the anti-LMW mAbs recognize CD630Δ*erm* and are not cross-reacting with other *Clostridium* strains or species.

### LMW is expressed at the surface of vegetative forms, but not spores

SlpA is expressed in the proteome of *C. difficile* spores, but whether the protein is exposed on the surface of the spores remains unknown.^18^ Therefore, we analyzed by microscopy the binding of the anti-LMW mAb NF10, displaying the best K_D_ and highest MFI in the flow cytometry assay on whole bacteria, to spores as well as to the vegetative form of *C. difficile*. The NF10 stained the vegetative form but did not stain spores (Figure 2c), indicating that SlpA LMW is differentially exposed on the surface of *C. difficile* spores.

### *Anti-LMW mAbs enable* C. difficile *phagocytosis by neutrophils*

We next evaluated whether SlpA LMW was a suitable target enabling or increasing phagocytosis of *C. difficile* by neutrophils using SlpA LMW mAbs, as it might occur during CDI after epithelial breakdown by toxins secreted by *C. difficile* ^19^ and invasion of the intestinal villi by bacteria and neutrophils.^20^ We used a standard *in vitro* phagocytosis flow cytometric assay, in which fluorescent dye-labeled bacteria were opsonized with anti-bacterial IgG mAbs and incubated with purified human neutrophils. All anti-LMW mAbs, being of the mouse IgG1 isotype, interacted with human IgG receptors (FcγRs)^21^ expressed by human neutrophils. Three out of the five high affinity binding anti-LMW mAbs enabled neutrophil-dependent phagocytosis of *C. difficile* (Figure 2d). Surprisingly, we found no correlation between phagocytosis and staining by flow cytometry with mAb KH2, which exhibited strong binding to *C. difficile* but resulted in minimal phagocytosis. In contrast, mAb 2B7 strongly induced phagocytosis, suggesting a unique property of mAb 2B7 to favor phagocytosis. Because of their low affinity binding to SlpA, mAbs 4G4 and 2C4 were excluded from the analysis.

Altogether, these results demonstrate that anti-LMW mAbs generated in this study recognize *C. difficile* in a vegetative state and enhance phagocytosis by neutrophils.

### C. difficile *growth is inhibited solely by mAb NF10*

**Figure 3.**
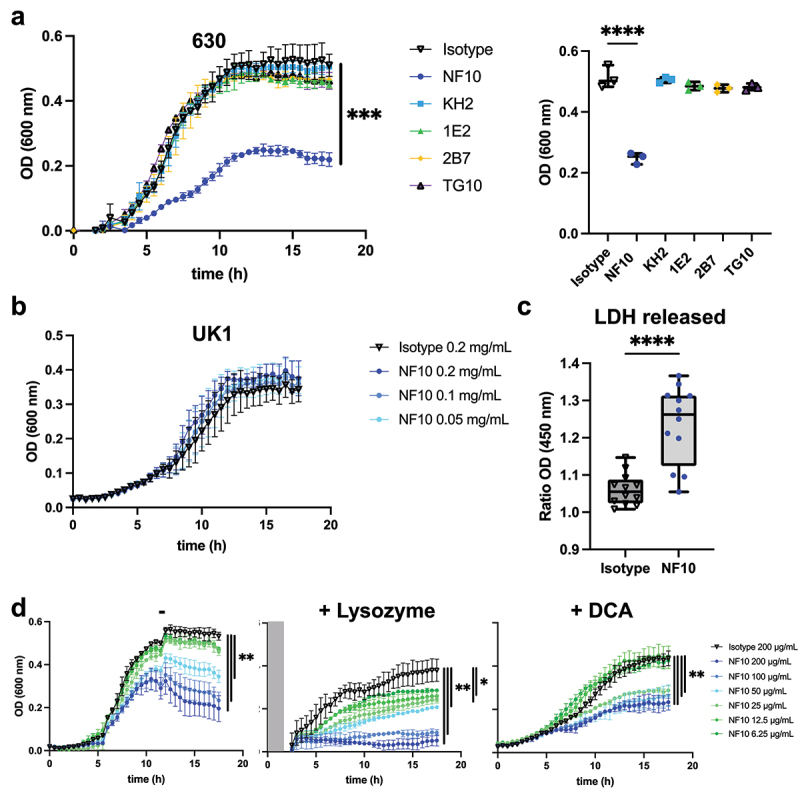
Effect on growth of anti-LMW mAbs and sensitivity to lysozyme and DCA. Cultures of *C. difficile* 630Δerm were inoculated at an OD_600 nm_ of 0.05 and grown anaerobically at 37°C with OD_600 nm_ measurements every 30 min. a. Effect of anti-LMW mAbs was assessed on growth. Left panel represents growth curves until 18 h with measurements every 30 min for all anti-LMW mAbs and isotype. Right panel represents quantitative analysis at 13 h for all anti-LMW mAbs and isotype. b. Effect of NF10 mAb was assessed on *C. difficile* UK1 strain growth at different concentrations. Data are presented as means and standard deviations from three technical replicates. c. LDH activity in the supernatant was normalized to condition without antibodies. The interquartile boxplots show medians (middle line), and the whiskers indicate minimal and maximal values. Asterisks indicate statistical significance calculated with a one-way ANOVA test followed by a Dunnett’s multiple comparison test (****p <.0001). Experiments were performed with two biological replicates in six technical replicates. d. Cultures of *C. difficile* 630Δerm incubated with different concentrations of NF10 mAb were monitored in combination with lysozyme (500 μg/ml), which was added after 2.5 h growth or DCA (240 µM). Isotype control (dark lines) was included in all experiments. Data are presented as mean values (±SD) from three technical replicates. Asterisks indicate statistical significance with a two-way ANOVA test (ns: not significant; *p <.05, **p <.01, *** *p* <.001, and ****p <.0001).

The S-layer is essential for *C. difficile* fitness, as *de novo* S-layer proteins are assembled during cell growth and division.^3^ We therefore investigated whether targeting SlpA LMW might affect bacterial growth. MAb NF10 strongly impacted *C. difficile* growth in suspension, which only reached ~ 50% of the plateau at 13 h of culture when compared to that in the presence of an isotype control mAb (Figure 3a). A minimum concentration of 50 µg/mL of the mAb was necessary to detect a statistically significant effect on growth (Supplemental Figure 1d). The effect of mAb NF10 was specific for the *C. difficile* strain CD630Δ*erm* because it did not affect the growth of the *C. difficile* UK1 strain belonging to ribotype 027 (Figure 3b). None of the other anti–LMW mAbs had any measurable effect on bacterial growth.

These results underline the unique ability of mAb NF10 to inhibit the growth of *C. difficile* strain CD630Δ*erm*.

### Bacterial lysis is promoted by mAb NF10

Next, we sought to determine the molecular mechanism underlying the inhibitory effects of the anti-LMW mAb NF10 on *C. difficile* growth. A pool of SlpA precursors was reported to be localized within the bacterial cell wall, available to repair openings in the S-layer during cell growth or damage.^22^ Thus, we hypothesized that the NF10 mAb could affect SlpA renewal in the S-layer, thereby promoting bacterial lysis. To quantify cell lysis during the exponential growth phase in the presence of NF10 mAb, we monitored the release of lactate dehydrogenase (LDH), a strictly cytoplasmic enzyme used to determine cell viability.^23^ We found significantly more LDH in the supernatants of NF10-exposed than in isotype control-exposed bacterial cultures (Figure 3c), supporting the hypothesis that the NF10 mAb weakens the integrity of the bacterial membrane.

If bacterial membrane integrity is compromised, it should become vulnerable to enzymes, particularly lysozyme, a protein produced by Paneth cells in the small intestine and ascending colon in humans. *C. difficile* wild-type strains are highly resistant to lysozyme, whereas the growth of *C. difficile* SlpA mutants is strongly affected in the presence of lysozyme.^5^ Strikingly, whereas high concentrations of NF10 (100 and 200 μg/mL) only partially inhibited the growth of *C. difficile* under standard culture conditions, it totally arrested bacterial growth in the presence of lysozyme (Figure 3d, Supplemental Fig. S1e). Moreover, low concentrations of NF10 (6.25 μg/ml to 25 μg/mL) that did not affect growth under standard culture conditions significantly inhibited growth in the presence of lysozyme. Secondary bile acid deoxycholate (DCA) plays a major role in CDI^24^ and can abrogate the growth of *C. difficile* at high doses (i.e., 25 µg/mL).^25^ The addition of mAb NF10 significantly inhibited growth of *C. difficile* in the presence of subinhibitory concentrations of DCA,^26^ even at concentrations of mAb insufficient to inhibit growth in standard culture conditions (Figure 3d, Supplemental Fig. S1f).

Together, these results show that mAb NF10 potentiates the detrimental effect of lysozyme or bile acid on *C. difficile* growth in a synergistic manner.

### *Anti-LMW mAbs differentially alter toxin secretion by* C. difficile

**Figure 4.**
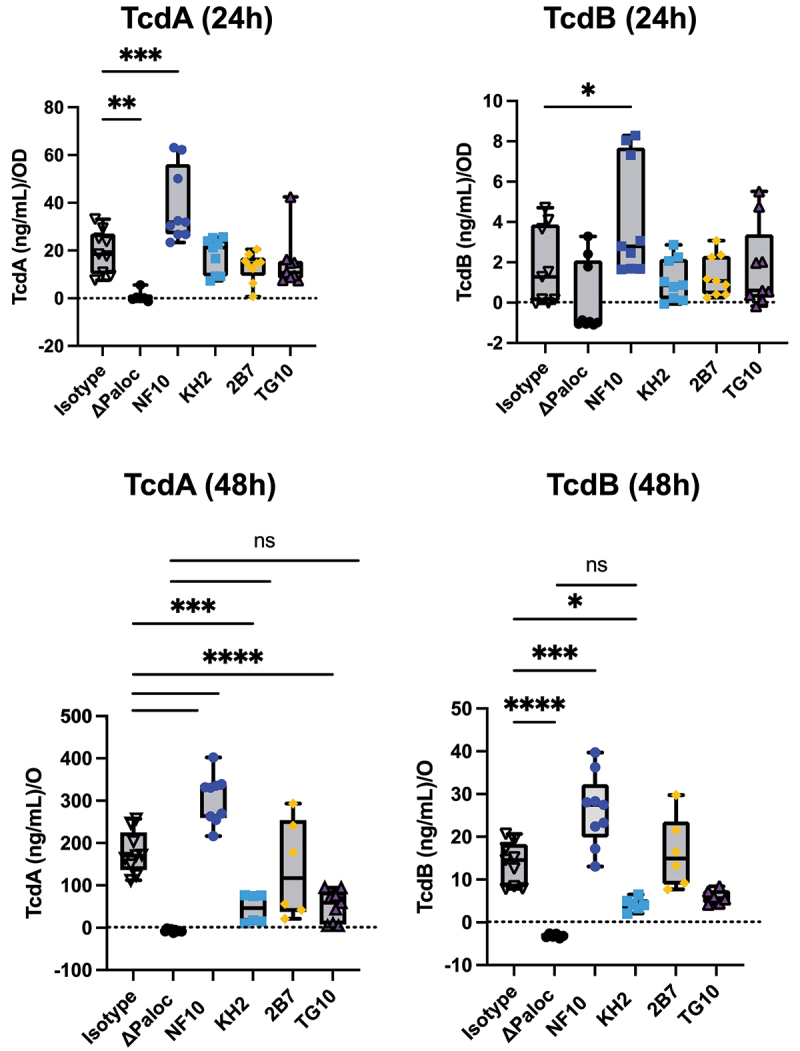
Anti-LMW mAbs modulate *C. difficile* toxin secretion. Quantification of TcdA or TcdB toxin secretion in CD630Δerm in the presence of anti-LMW mAbs or isotype control. CD630ΔermΔPaloc mutant strain has been tested as a negative control. Toxin titers in culture supernatants were determined at 24 h and 48 h by ELISA. Boxplots show medians (middle line) and interquartile range, and the whiskers indicate minimal and maximal values of three replicates. Asterisks indicate statistical significance calculated with a one-way ANOVA test followed by a Dunnett’s multiple comparison test (ns: not significant; *p <.05, * *p* <.01, ***p <.001, and ****p <.0001).

*C. difficile* secrete toxins through pores in the S-layer by a mechanism still incompletely known.^4^ Given that impaired toxin production has been reported in *C. difficile* SlpA-null mutants,^5^ we explored whether anti-LMW mAbs were able to alter bacterial toxin secretion *in vitro*. Under steady culture conditions, CD630Δ*erm* secreted ~18 ng/mL at 24 h and ~170 ng/mL at 48 h of TcdA, and ~1 ng/mL at 24 h and ~14 ng/mL at 48 h of TcdB (Figure 4). As expected, the pathogenicity locus (Paloc)-deficient *C. difficile* mutant (ΔPaloc)^26^ lacking the toxin A (TcdA) and B (TcdB) genes did not secrete any detectable quantities of either toxin. Incubation with mAb NF10, but not with any of the other anti-LMW mAbs, significantly increased TcdA and TcdB secretion at both 24 h and 48 h. In contrast, the mAbs KH2 and TG10 significantly reduced TcdA and TcdB secretion at 48 h. Surprisingly, mAb 2B7, which partially recognizes the same epitope on SlpA as mAb KH2 (Figure 1d) and displays a higher affinity for LMW (Table S1), did not affect the secretion of either toxin.

Together, these results indicate that even though anti-LMW mAbs NF10, KH2, and TG10 bind to the same target on the *C. difficile* surface, they induce contrasting effects on toxin secretion that appear to be epitope-dependent.

### *Anti-LMW mAbs NF10 and 2B7 increase* C. difficile *biofilm formation*

*C. difficile* CWP84 mutants with an altered S-layer were reported to increase the biomass of their biofilm, as compared to the parental strain, suggesting a role for SlpA in *C. difficile* biofilm formation.^9^ Therefore, we assumed that biofilm formation could be modulated when the *C. difficile* S-layer is constrained by anti–LMW mAbs.

**Figure 5.**
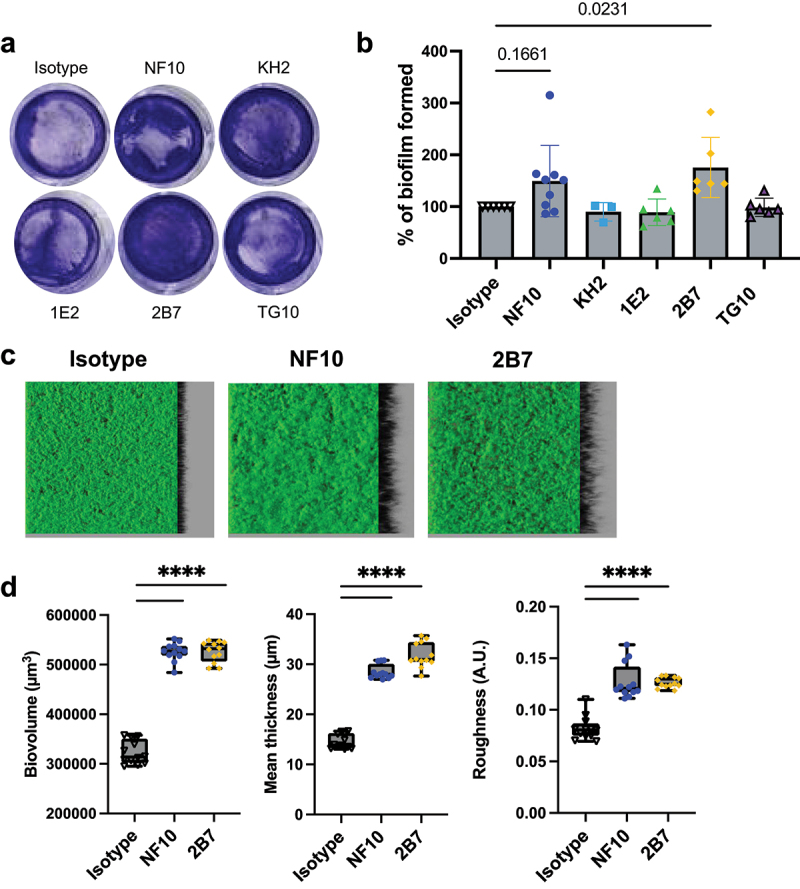
Anti–LMW mAbs influence *C. difficile* biofilm formation. Biofilm formation with CD630Δ*erm* strain was assayed in BHISG medium supplemented with 240 µM DCA. a. Representative pictures of biofilm formed in the presence of indicated mAbs after crystal violet staining. b. Biofilm biomass was assessed by absorbance at 600 nm. Histograms show medians (middle line) and whiskers indicate standard deviation of at least three independent experiments. c. visualization mAbs-coated CD630Δ*erm* biofilms stained with SYTO9. Z-stacks were analyzed with BiofilmQ. CLSM images are representative of three independent biological replicates. For each image, the virtual shadow projection of the biofilm is shown in dark on the right. d. Quantitative analyses were performed with BiofilmQ to measure the biovolume, thickness and roughness of the biofilms. The interquartile boxplots show medians (middle line) and the whiskers indicate minimal and maximal values of three replicative samples. Asterisks indicate statistical significance with a one-way ANOVA test followed by a Dunnett’s multiple comparison test (****p <.0001).

In the presence of sub-inhibitory concentrations of DCA, the CD630Δ*erm* strain formed a biofilm which was found to significantly increase in result of the addition of mAb 2B7 to the culture, whereas a similar but non-significant trend was observed with mAb NF10 (increase in biofilm formation compared to the wild type 100%):175%, *p* =.0231 and 149%, *p* =.1661, for 2B7 and NF10, respectively; Figure 5b).

Analysis of biofilm volume, thickness, and roughness (i.e., unevenness of the biofilm surface) by confocal laser scanning microscopy showed that incubation of *C. difficile* cultures with either NF10 or 2B7 induced a ~ 1.7-fold increase in biovolume, a ~ 2-fold increase in thickness, and a ~ 1.6-fold increase in roughness when compared to DCA-induced biofilms in the absence of mAbs (Figure 5c,d).

These results highlight the contribution of SlpA LMW to biofilm formation, with epitope-dependent enhancement of biofilm generation revealed by the two anti–LMW mAbs.

## Discussion

*C. difficile* is a complex anaerobic pathogen to study, because of the lack of relevant tools, in particular antibodies, to investigate the contribution of its surface components to its pathogenic properties. In the present study, we identified a series of mAbs with different binding affinities for SlpA which were used to investigate the contribution of LMW to growth, toxin secretion and biofilm formation, as well as its potential as a target for neutrophil-dependent phagocytosis. These anti–LMW mAbs were found to have differential, and even opposite, effects on the biological properties of *C. difficile* depending on the epitope recognized on SlpA. The high-affinity mAb NF10 had multiple effects on *C. difficile* by impairing growth in a dose-dependent manner, increasing susceptibility to lysis by lysozyme and bile acids, and affecting toxin secretion and biofilm formation. No such effect was observed with the anti–LMW mAbs KH2 and TG10. However, contrary to mAb NF10, the latter mAbs inhibited toxin secretion, suggesting an epitope-dependent regulation of *C. difficile* biology by the low-molecular-weight subunit of SlpA

An unexpected feature of these anti–LMW mAbs is their contrasting effect on *C. difficile* physiology depending on the epitope to which they bind. Antibodies and nanobodies targeting *C. difficile* S-layer have been proposed as potential therapeutic agents.^16,27^ Likewise, active and passive immunization strategies have been tested with varying degrees of success in preventing or treating CDI.^15,28^ Our findings suggest that anti-S-layer humoral responses have both beneficial and detrimental effects. Thus, the precise definition of the epitopes recognized by the S-layer associated with their effect on various *C. difficile* functions is of importance for successfully designing anti-S-layer therapeutic mAbs. Furthermore, even if a toxin-suppressing antibody might at first glance appear beneficial to the host, it might also facilitate biofilm formation and, consequently, promote gut persistence of *C. difficile*. Our data prompted us to test novel therapeutic agents not only in single-episode CDI models, but also in recurrence models, to evaluate the role of biofilms as a reservoir for further infections.

The S-layer is an important component involved in bacterial growth because in dividing cells a new S-layer must be continuously assembled. Although no previous study has evaluated the effect of targeting the *C. difficile* S-layer owing to the lack of specific antibodies, a related study on *Bacillus anthracis* showed that anti-S-layer nanobodies attenuated bacterial growth,^29^ reminiscent of the inhibitory effect of mAb NF10 on the growth of *C. difficile*. The authors showed that nanobodies inhibited S-layer *de novo* assembly with full dissolution of the S-layer polymers, which resulted in drastic morphological defects and S-layer disruption.^29^ Similarly, mAb NF10 is likely to interfere with optimal S-layer compaction, leading to morphological defects and bacterial lysis. In contrast, *C. difficile* S-layer null mutants reportedly do not show any growth defects but are more susceptible to the lytic effects of lysozyme and antimicrobial peptides such as LL-37^5^ thereby corroborating the notion that the *C. difficile* S-layer forms a tightly compact barrier around the bacteria which is normally impenetrable to large molecules.^4^ In this respect, the addition of mAb NF10 was found to increase the susceptibility of *C. difficile* to lysozyme. We therefore propose that, in addition to its capacity to cause S-layer disruption, mAb NF10 might facilitate transport of large molecules across the cell membrane, such as lysozyme, following its interaction with *C. difficile* LMW, a property that holds promise for the use of this mAb for specific drug delivery.

Toxin secretion is a major physiological process that confers pathogenicity to *C. difficile*. Because CDI symptoms are mainly due to the production of TcdA and TcdB, the regulation of their expression and the mechanisms involved in their secretion have been extensively studied.^30^ Toxin synthesis is growth phase-dependent and is regulated in response to a variety of environmental factors, such as the availability of specific nutrients, temperature, and cell density.^30–33^ While toxin secretion depends on a holin-dependent system,^31^ how toxins cross the *C. difficile* membrane and, consequently, how they interact with the S-layer without bacterial lysis remains an open question.^4^ The S layer must create discrete pores to allow toxin export while maintaining bacterial integrity.

Interestingly, three of the anti–LMW mAbs differentially altered toxin secretion by either increasing or decreasing it, pointing toward a dual role of the S-layer in toxin release. On the one hand, S-layer disruption by mAb NF10 may lead to a massive toxin release, on the other hand mAbs KH2 and TG10 may “rigidify” or “lock” the S-layer, thus suppressing toxin export. Consistent with our findings, mutants affecting *C. difficile* S-layer were reported to display these contrasting features as well.^5,9,34^ We also hypothesize that changes in the integrity of the S-layer may modulate toxin expression. Further functional and structural studies are needed to determine how SlpA affects transmembrane import-export mechanisms *in C. difficile*.

Another aspect of *C. difficile* pathogenicity is its ability to form biofilms, which has been suggested to contribute to its pathogenesis and persistence.^35^ Biofilm-like structures have been observed in CDI mouse models *in vivo* .^36,37^ Analysis of *C. difficile* biofilm composition showed that extracellular DNA is an essential component of the biofilm matrix. Notably, incubation with DNase I drastically reduced the biofilm biomass,^38,39^ which corroborates our hypothesis that mAb NF10-induced lysis facilitates biofilm formation by increasing the amount of extracellular DNA and proteins in the biofilm matrix. Beyond S-layer disruption and bacterial lysis, the extent to which S-layer proteins, such as LMW, are *per se* involved in biofilm formation remains unclear. Inhibition of S-layer-mediated aggregation could also impact the early steps of biofilm formation, as has been demonstrated for *Lactobacillus helveticus M92* .^40^

Our study has several limitations. We studied biofilm formation and architecture in a closed system using only one *C. difficile* strain. A recent study demonstrated that biofilms grown in tissue culture plates and biofilms obtained in open systems harbor different characteristics in terms of cell-surface protein expression^41;^ therefore, it would be judicious to evaluate anti–LMW mAbs in other biofilm-forming conditions. It is of note that the biofilm-forming ability differs between *C. difficile* strains,^42^ making it difficult to assign a single model to all ribotypes. Moreover, knowing the precise LMW epitopes that are recognized by the mAbs described in this study could help decipher their varying impact on *C. difficile* physiology. Secretory IgA has been reported to shape functional microbial fitness, depending on the recognized antigen and epitopes.^43^ The absence of the D2 domain of the LMW in *C. difficile* has been shown to be sufficient to confer susceptibility to lysozyme, thereby indicating its crucial role in maintaining S-layer integrity.^4^ We hypothesize that mAb NF10 might interact with an epitope in the D2 domain, thus impairing its function and mimicking mutants lacking this domain.

In conclusion, we demonstrate herein that targeting the S-layer of *C. difficile* with SlpA-specific mAbs has several contrasting effects on the physiology of this bacterial species. Characterization of the epitope(s) that these mAbs recognize may provide ways to interfere with the involvement of the S-layer of *C. difficile*, such as inhibiting bacterial growth or toxin secretion, which could lead to novel therapeutic strategies for the treatment of CDI.

## Methods

### Production of recombinant LMW proteins

Recombinant *C. difficile* LMW-630 was produced as a C-terminal 6×His-tagged protein from plasmid pET-28a(+) (Twist Biosciences, #69864). Plasmids were transformed into *Escherichia coli* strain D43 and grown in NZY auto-induction lysogeny broth (LB) medium (#MB180; NZYtech). Bacteria were harvested by centrifugation and lysed using the Precellys system, according to the manufacturer’s instructions (#P002511-PEVT0-A.0). Recombinant LMW-SLP proteins from the soluble fraction were purified using affinity chromatography on Ni-agarose columns using AKTA Prime (GE Healthcare, #11001313). The proteins were dialyzed against 10 mM HEPES (pH 7.5) and 150 mM NaCl prior to analysis or long-term storage.

### *Generation of mAbs against LMW of* C. difficile *strain 630*

Knock-in mice expressing human antibody variable genes for the heavy (VH) and kappa light chain (Vκ) (VelocImmune, Supplemental Fig. S1a) were previously described^44,45^ and provided by Regeneron Pharmaceuticals to be bred at Institut Pasteur. BALB/c mice were purchased from Janvier Labs. All animal care and experimental procedures were conducted in compliance with national guidelines. The study, registered under #210111, was approved by the Animal Ethics Committee of CETEA (Institut Pasteur, Paris, France) and by the French Ministry of Research.

BALB/c and VelocImmune mice were injected intraperitoneally on days 0, 21, and 42 with 50 μg of recombinant LMW630 mixed with 200 ng/mouse pertussis toxin (Sigma-Aldrich, MO, USA). An enzyme-linked immunosorbent assay was performed to measure serum responses to antigens (see methods below), and the three immunized animals with the highest serum titers were boosted with the same preparation. Four days later, splenocytes were fused with myeloma cells P3X63Ag8 (ATCC, France) using a ClonaCell-HY Hybridoma Kit, according to the manufacturer’s instructions (StemCell Technologies, Canada). Culture supernatants were screened using ELISA (see below), and antigen-reactive clones were expanded in serum IgG-free RPMI-1640 (Sigma-Aldrich) into roller bottles at 37°C. After 14 days, the supernatants were harvested by centrifugation at 2500 rpm for 30 min and filtered through a 0.2 µm filter. Antibodies were purified by Protein A affinity chromatography (AKTA, Cytiva, Germany), as described previously.^46^

### ELISA assays

Maxisorp microtiter plates (Dutscher, France) were coated with 0.3 μg LMW630 recombinant protein in carbonate buffer (Na_2_CO_3_/NaHCO_3_) for 2 h at room temperature (RT). Free sites were blocked by a 2-hour incubation at RT with 1X-PBS 1% BSA. Plates were washed three times with 1X-PBS 0.05% Tween 20 (PBS-T) before being co-incubated with serum, supernatants, or mAbs at different concentrations (from 10^−6^ μg/mL to 10 μg/mL) for 1 h at RT. After five washes, HRP-conjugated goat anti-mouse IgG Heavy and Light Chain antibody (Bethyl, TX, USA; dilution 1:20,000) was added for 1 h at RT, followed by incubation with OPD substrate for 10 min (Sigma-Aldrich, MO, USA). Absorbance was measured at 495 *vs* 620 nm on an ELISA plate reader (Berthold, France).

### Bio-layer interferometry

Biolayer interferometry assays were performed using Anti-Mouse IgG Fc Capture biosensors (18–5088) in an Octet Red384 instrument (ForteBio, USA). MAbs (10 μg/mL) were captured on the sensors at 25°C for 1,800 s. The biosensors were equilibrated for 10 minutes in 1X-PBS, 0,05% Tween 20, and 0.1% BSA (PBS-BT) prior to measurement. Association was monitored for 1,200s in PBS-BT with LMW630 at concentrations ranging from 0.01 nM to 500 nM, followed by dissociation for 1,200s in PBS-BT. For epitope competition assays, the sensors were further immersed in solutions containing mAb at 10 μg/mL. Biosensor regeneration was performed by alternating 30s cycles of regeneration buffer (glycine HCl, 10 mM, pH 2.0) and 30s of PBS-BT for three cycles. Traces were reference sensors (sensors loaded with an irrelevant mAb) subtracted, and curve fitting was performed using a global 1:1 binding model in the HT Data analysis software 11.1 (ForteBio, USA), allowing the determination of K_D_ values.

### IgH and IgL sequencing

Total RNA was extracted from murine splenocytes using a NuceloSpin RNA Plus Kit (Macherey-Nagel, France), according to the manufacturer’s instructions. cDNA was generated at 50°C for 60 min using random primers and SuperScript III Reverse Transcriptase (Invitrogen). The primer pairs for IgH and IgL described in Supplemental Table S2 were used for amplification with GoTaq G2 polymerase (Promega, WI, USA). Amplification was performed using 35 cycles of PCR, each consisting of 94°C for 30 s, 63°C for 30 s, and 72°C for 30 s. At the end of the 35 cycles, the samples were run for an additional 10 min at 72°C and analyzed by 1.5% agarose gel electrophoresis. The PCR products were then sequenced by Eurofins (France) using 3’-primers.

### Flow cytometry assay

The binding of mAbs to whole bacteria was assessed using bacterial flow cytometry assays, as previously described.^17^ Briefly, fixed *C. difficile* cells (10^6^/condition) were stained with 5 μM SYTO9 dye (Thermo Fisher Scientific, MA, USA) in 0.9% NaCl for 30 min at RT. Bacteria were washed (10 min, 4,000 g, 4°C) and resuspended in 1X PBS, 2% BSA, and 0.02% Sodium Azide (PBA). Mabs were pre-diluted in PBA at 20 µg/mL and incubated for 30 min at 4^◦^C. Bacteria were washed, and incubated with AF647 AffiniPure goat anti-mouse IgG (H+L) antibody or isotype control (dilution 1:200, Jackson ImmunoResearch, PA, USA) for 30 min at 4^◦^C. After washing, bacteria were resuspended in sterile 1X-PBS. Flow cytometry acquisition was performed on a MacsQuant cytometer (Miltenyi, Germany) and analyzed using FlowJo software (BD Biosciences, CA, USA).

### Isolation of human neutrophils

Human peripheral blood samples were collected from healthy volunteers using ethylenediaminetetraacetic acid. Blood neutrophils were separated by negative magnetic selection (MACSxpress, Miltenyi Biotec, Germany), according to the manufacturer’s instructions. After negative selection, the neutrophil-enriched suspension was recovered and residual erythrocytes were removed using the MACSxpress Erythrocyte Depletion kit (Miltenyi Biotec, Germany). The resulting neutrophil suspension was washed with HBSS (Sigma-Aldrich, MO, USA) and resuspended to an appropriate volume in HBSS (Ca^2+^/Mg^2+^) + 2% fetal calf serum (Cytiva, Germany).

### Phagocytosis assay

Human neutrophils were plated at a density of 8 × 10^5^ cells/ml. Fixed *C. difficile* was incubated with one mAb at 20 µg/mL or a cocktail of mAbs NF10, KH2, 1E2, 2B7, and TG10 at an equimolar ratio and stained with pHRodo dye (Thermo Fisher Scientific) following the manufacturer’s instructions. An irrelevant mouse IgG (in-house produced) was used as an isotype control. Bacteria were then incubated with neutrophils at a Multiplicity Of Infection (MOI) of 100 for 1.5 h at 37°C (20,000 neutrophils for each condition). Flow cytometry acquisition was performed on a MacsQuant16 cytometer (Miltenyi, Germany) and analyzed using FlowJo software v10.8.1 (BD Biosciences, CA, USA).

### Bacterial strains and culture conditions

*C. difficile* 630Δerm,^47^ a spontaneous erythromycin-sensitive derivative of the reference strain 630, and *C. difficile* strain UK1^48^ of ribotype 027 strains were grown anaerobically (5% H2, 5% CO2, 90% N2) in TY medium (30 g/L tryptone, 20 g/L yeast extract) or in Brain Heart Infusion (BHI) medium supplemented with 0.5% (w/v) yeast extract, 0.01 mg/mL cysteine, and 100 mM glucose (BHISG). All media and chemicals were purchased from Sigma-Aldrich.

### Growth assays, lysozyme resistance and quantification of lysis

Overnight *C. difficile* cultures were grown in TY broth, subcultured to an Optical Density at 600 nm (OD_600 nm_) of 0.05 in 200 µL of BHISG or, when appropriate, BHISG supplemented with DCA (240 µM, Sigma-Aldrich) in 96-well flat-bottom plates (Merck, Germany) and then grown for 24 h or 18 h with OD_600 nm_ measurements taken every 30 min by GloMax Plate Reader (Promega, WI, USA). Anaerobiosis was maintained using an O_2_-less sealing film (Sigma-Aldrich). Where appropriate, lysozyme (1 mg/mL) was added after 2.5 h of growth. Experiments were performed at least in triplicates. For lysis quantification, LDH was measured in 13 h-culture supernatants using the CytoTox 96 Non-Radioactive cytotoxicity assay according to the manufacturer’s instructions (Promega).

### Biofilm assays

Overnight cultures of *C. difficile* 630Δerm grown in TY medium were diluted 1:100 in fresh BHISG supplemented or not supplemented with 240 µM DCA and 0.2 mg/mL mAbs. Diluted cultures (1 mL) were added to 24-well plates (polystyrene tissue culture-treated plates; Costar, USA). The plates were incubated at 37°C in an anaerobic environment for 48 h. Biofilm biomass was measured using an established method.^25^ Briefly, the biofilms were washed with 1X-PBS and stained with crystal violet for 5 min. After washing, crystal violet was resuspended in ethanol, and the OD_600 nm_ was measured.

### Confocal Laser Scanning Microscopy (CLSM)

Biofilms were grown in 96-well plates (Microclear, Greiner Bio-one, France) in BHISG supplemented with DCA (240 μM) and anti–LMW630 mAbs, as described above. After 48 h, the supernatants were carefully removed by pipetting, and the biofilms were fixed with 4% paraformaldehyde (Sigma-Aldrich). The biomass was then stained with SYTO9 dye (Life Technologies, USA) at a final concentration of 20 μM. The dye was incubated for 30 min before the CLSM imaging and analysis. Z-stacks of horizontal plane images were acquired in 1 μm steps using a Leica SP8 AOBS inverted laser scanning microscope (CLSM, LEICA Microsystems, Wetzlar, Germany) on the INRAE MIMA2 platform. At least two stacks of images were acquired randomly on three independent samples at 800 Hz with a ×63water objective (N.A. = 1.2). Fluorophores were excited, and their emissions were captured as prescribed by the manufacturer.

### Analysis of CLSM biofilm images

Z-stacks from the CLSM experiments were analyzed using BiofilmQ software^49^ to extract quantitative geometric descriptors of the biofilm structures. The images were treated using the same process in each fluorescence channel. First, the images were denoised by convolution (dxy = 5 and dz = 3), and then segmented into two classes using the OTSU thresholding method with a sensitivity of 2. The detected signal was then declumped in 3.68 μm cubes and small objects were removed with a threshold of (0.5 μm^3^) to clean the remaining noise. The exported data were analyzed using the software Imaris to generate biofilm 3D projections and GraphPad Prism to generate quantitative graphs.

### Toxin a & B assays

*C. difficile* 630Δerm and 630ΔermΔPaloc were grown in 6-well plates containing 2 mL TY medium for either 24 h or 48 h with 0.2 mg/mL of anti–LMW mAbs when specified. Absorbance was measured at 600 nm, and the cultures were harvested and centrifuged at 4,000 rpm for 5 min. Toxins were assessed in the supernatants using ELISA. Maxisorp microtiter plates (Dutscher, France) were coated with 5 μg/mL anti-TcdB capture antibody (BBI Solutions, Madison, WI) or anti-TcdA capture antibody (Novus Biological, CO, USA). Purified toxins A and B (Sigma-Aldrich) were used as the standards. The supernatants were added for 1h30 at RT. After washing, the anti-toxin B biotinylated antibody (BBI solutions, Madison, WI, USA) followed by high-sensitivity streptavidin-HRP conjugate (ThermoFisher, Waltham, MA), or anti-toxin A HRP-conjugated antibody (LSBio, WA, USA) signal was detected with TMB substrate (ThermoFisher, Waltham, MA) at 450 nm using an ELISA plate reader (Berthold, France). Toxin concentrations were normalized to the OD_600 nm_ values for each well.

### Statistical analysis

Growth, LDH, toxin, and biofilm assay values were analyzed using GraphPad Prism 8.0 (GraphPad, San Diego, CA). Statistical analysis was performed using one-way ANOVA variance followed by Dunnett’s multiple comparison test. Statistical significance was set at *p* value ≤.05.

## Supplementary Material

Supplemental Material

## Data Availability

The authors confirm that data supporting the findings of this study are available within the article and its supplementary materials.
