# Supplementary material for: Anti-S-layer monoclonal antibodies impact Clostridioides difficile physiology
Source: Gut Microbes. 2024 Jan 30;16(1):2301147. doi: 10.1080/19490976.2023.2301147 (PMC10829821; doi:10.1080/19490976.2023.2301147)
Supplement: Supplemental Material [file KGMI_A_2301147_SM9515.zip › supp figure 1 revised.docx]

**Supplemental Figure 1**

**a**

**Mouse IgH constants & controlling regions**

**V_H_**

**D_H_**

**J_H_**

**μ/δ γ3 γ1 γ2b γ2a ε α**

**Eμ**

**3’RR**

**Humanized IgH variable regions**

**Mouse IgK constant & controlling regions**

**V_κ_**

**J_κ_**

κ

**iEκ**

**Humanized IgK variable regions**

**3’Eκ**

**VelocImmune® mice**

**Human V_λ_**

**Human J_λ_**

**Mouse C_λ_1**

IgH locus

IgK locus

**Mouse IgH constants & controlling regions**

**V_H_**

**D_H_**

**J_H_**

**μ/δ γ3 γ1 γ2b γ2a ε α**

**Eμ**

**3’RR**

**Humanized IgH variable regions**

**Mouse IgK constant & controlling regions**

**V_κ_**

**J_κ_**

κ

**iEκ**

**Humanized IgK variable regions**

**3’Eκ**

**VelocImmune® mice with a LiK allele**

IgH locus

IgK locus

**Mouse IgL constant & IgK controlling regions**

**V_λ_**

**J_λ_**

λ1

**iEκ**

**Humanized Igλ variable regions**

**3’Eκ**

KoK allele

LiK allele

**+ DCA**

**+ Lysozyme**

**-**

**d**

**e**

**f**


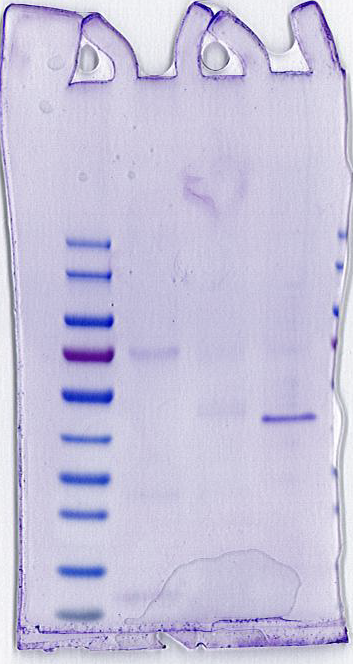


55

70

40

35


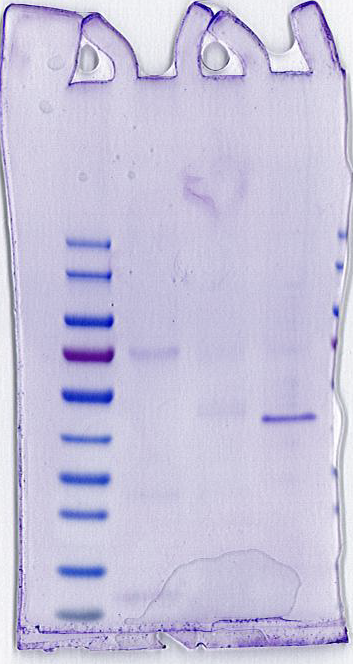


**b**

**c**

LMW

55

70

100

130


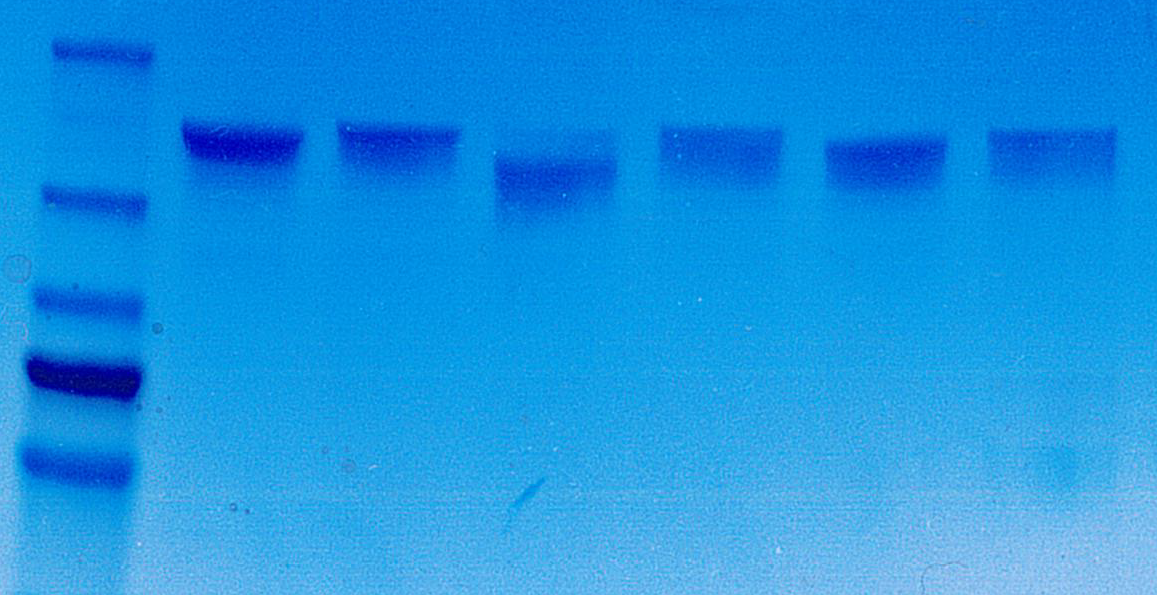


250

NF10

KH2

1E2

2B7

2C4

4G4
