# Supplementary figures and images for: Anti-S-layer monoclonal antibodies impact Clostridioides difficile physiology
Source: Gut Microbes. 2024 Jan 30;16(1):2301147. doi: 10.1080/19490976.2023.2301147 (PMC10829821; doi:10.1080/19490976.2023.2301147)

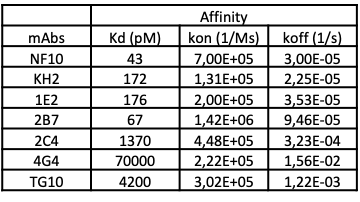


**Supplemental Table 1**

Supplement: Supplemental Material [file KGMI_A_2301147_SM9515.zip › Sup Table 1.docx]

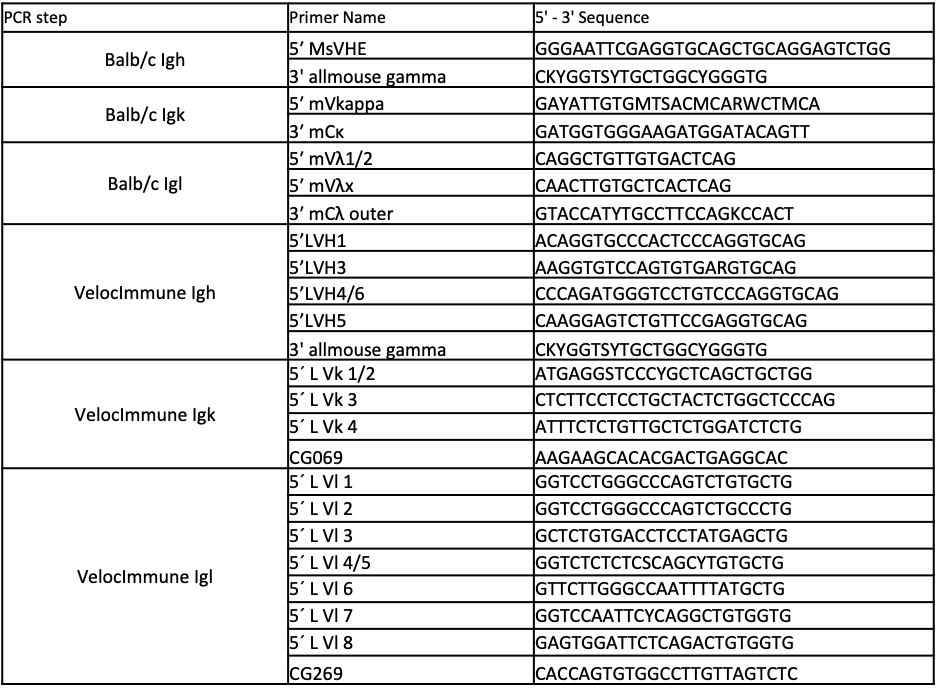


**Supplemental Table 2**

Supplement: Supplemental Material [file KGMI_A_2301147_SM9515.zip › Sup Table 2.docx]
